# Topiramate-Induced Fanconi Syndrome: A Case Report

**DOI:** 10.1016/j.xkme.2026.101522

**Published:** 2026-09-09

**Authors:** Daniela Berlinkova, Ria Rohatgi, Rajeev Rohatgi, Angela Z. Jiatu

**Affiliations:** 1 Department of Pharmacy, James J. Peters VAMC, Bronx, NY; 2 Harborfields High School, NY; 3 Icahn School of Medicine at Mount Sinai, NY; 4 Department of Medicine, James J. Peters VAMC, Bronx, NY

## Abstract

Topiramate (TPM), originally approved in 1996 for epilepsy, is now widely administered to prevent migraines, induce weight loss, and suppress alcohol cravings. Common renal adverse side effects include metabolic acidosis and nephrolithiasis; however, acute kidney injury (AKI) is uncommon or underreported. In this report, we present a case of a 41-year-old man with a history of hypertension, obesity, tonic-clonic seizures, and migraines who developed AKI associated with Fanconi syndrome while administered TPM 100 mg daily for migraine prevention. The Naranjo Adverse Drug Reaction Probability Scale, a measure of the likelihood that a drug is causing an adverse reaction, indicated that TPM probably (score of 8) caused AKI with Fanconi syndrome. After supportive therapy and drug cessation the patient’s kidney function normalized and the Fanconi syndrome resolved. This case is important for several reasons: (1) this is the third case of AKI and second case associated with Fanconi syndrome, which implies a relationship with TPM, (2) the cases illustrate that early recognition and discontinuation of TPM resolves AKI, and (3) common elements between the cases relay plausible risk factors. Thus, clinicians who prescribe TPM should consider monitoring patients for AKI and electrolyte abnormalities.

Topiramate (TPM) was originally marketed as an antiepileptic medication, but its use has expanded to include migraine prevention,^1^ weight loss,^2^ posttraumatic stress disorder,^3^ and suppress alcohol craving.^4^ Because of the multitude of uses, ∼10 million prescriptions of TPM have been written annually since 2019. TPM is not benign and can cause several adverse renal side effects, including metabolic acidosis and nephrolithiasis, but we report a second case of Fanconi syndrome and acute kidney injury (AKI) related to TPM.

Fanconi syndrome, or proximal tubular dysfunction, presents with urinary losses of amino acids, low-molecular weight protein, bicarbonate, glucose, phosphate, and sodium (Na).^5^ Fanconi syndrome is inherited, acquired by exposure to toxic agents, or idiopathic.^5^ Historically several classes of medications induce an acquired Fanconi syndrome: aminoglycosides, alkylating agents, valproic acid, aspirin, platinum agents, and antivirals.^6^ To date, a single case of TPM-related AKI with Fanconi syndrome has been reported.

### Case Report

A 41-year-old man with a medical history of hypertension, obesity, posttraumatic stress disorder, migraines, and tonic-clonic seizures presented to the emergency department (ED) with severe hyponatremia and AKI. Two months before admission, he developed lethargy, nausea, vomiting, and diarrhea. Five days before presentation, an outpatient serum chemistry revealed a serum Na (SNa) of 116 mEq/L and serum creatinine (Scr) of 2.8 mg/dL (baseline 1.1 mg/dL). At the ED, the patient appeared to be a well-nourished obese male with blood pressure of 119/60 mm Hg, heart rate of 94 beats per minute, afebrile with a normal respiratory rate, normal oxygen saturation, and no skin rashes. He was alert and oriented but demonstrated slowed mentation and delayed responses to questioning. The remainder of the physical examination was unremarkable. His home medications included hydrochlorothiazide (HCTZ) 50 mg/lisinopril 40 mg combination tablet daily, buspirone 30 mg twice daily, levetiracetam 750 mg twice daily, pantoprazole 40 mg daily, zolmitriptan 5 mg as needed for headaches, minoxidil 2.5 mg daily, and sildenafil 100 mg as needed. He had started TPM 50 mg daily for headaches 18 months before admission, and it was raised to 100 mg daily 13 months before admission. The patient quit smoking 15 years ago and consumes alcohol 2-3 times weekly. He denied using herbal medications or recent exposure to intravenous contrast.

Initial laboratory evaluation in the ED confirmed severe hypo-osmolal hyponatremia (SNa 116 mEq/L; serum osmolality 258 mOsm/kg), AKI (Scr 3.0 mg/dL; Fig 1), and near normal serum glucose 111 mg/dL (Table 1). The urine Na was 25 mEq/L, urine osmolality 136 mOsm/kg, and urine creatinine 52 mg/dL, leading to a calculated fractional excretion of Na of 1.2%. Arterial blood gas analysis (pH 7.434/pCO_2_ 45.4 mm Hg/bicarbonate 29.7 mEq/L) revealed a compensated metabolic alkalosis. Hemoglobin A1C was 5.2% (normal); however, urinalysis demonstrated persistent 3+ glucosuria (or ∼500 mg/dL glucose) with an elevated urine pH of 7.0 and no leukesterase, white blood cells, or red blood cells, findings consistent with Fanconi syndrome without interstitial nephritis. Computed tomography of the brain was unremarkable.

**Figure 1:**
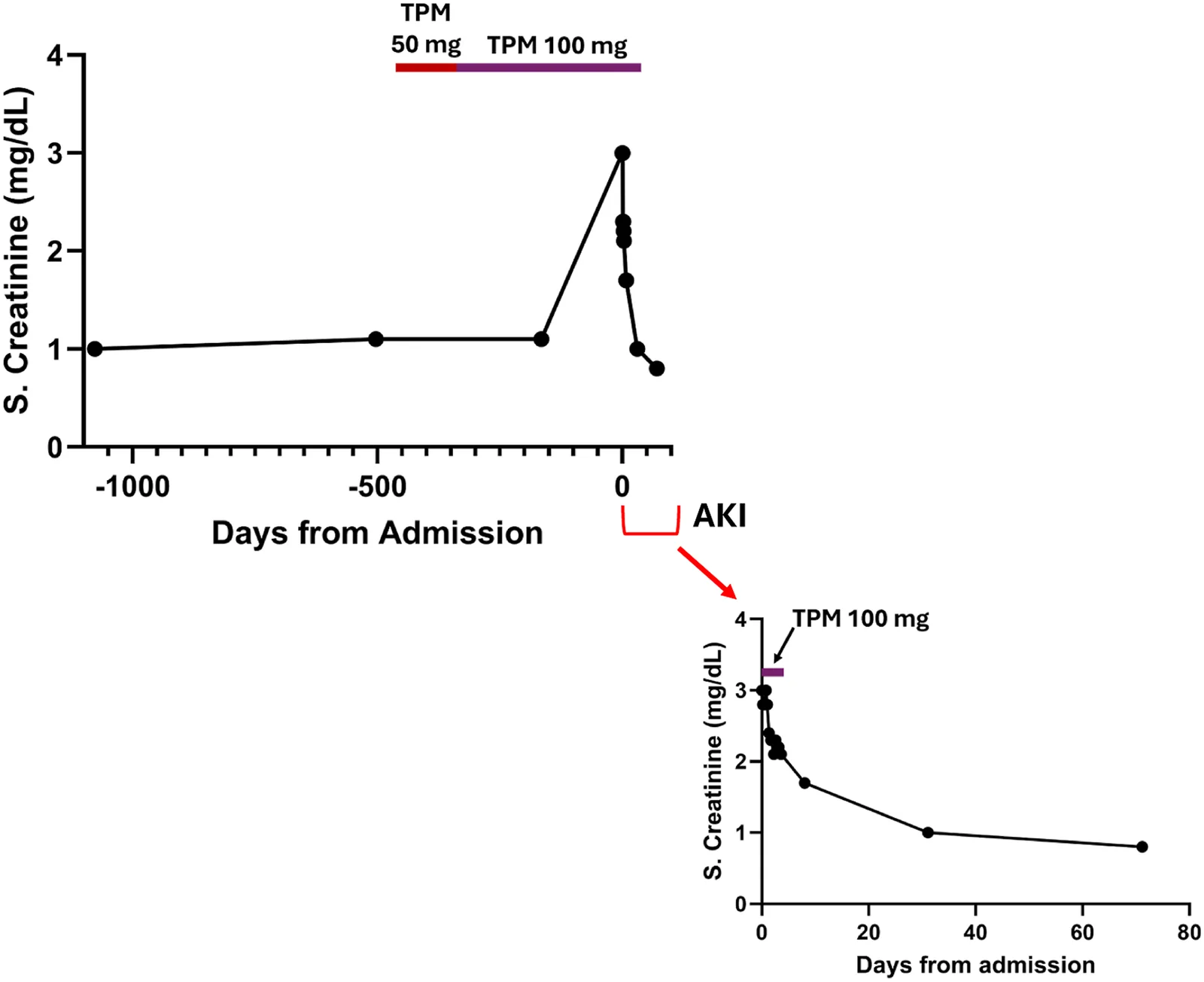
Changes in Scr in relation to TPM administration, and before and after hospitalization. The Scr is normal months before and after administering TPM to the patient; however, ∼13 months after starting a higher dose of TPM (day 0; hospitalization) the Scr increased acutely to 3.0 mg/dL. Post-AKI improvement in Scr is represented in the enlarged panel to the right. AKI, acute kidney injury; Scr, serum creatinine; TPM, Topiramate.

**Table 1:** The Change in Serum Chemistries Before, During, and After Stopping TPM

	18 Months Before TPM Initiation	1 month After TPM Initiation	7 Months After TPM Dose Increase	Admission and 11 Months After TPM Increase	Day 4 of Hospitalization, TPM Withdrawn After This Test	Day 5 of Hospitalization, TPM Discontinued for 1 Day	Day 4 Post Discharge, TPM Discontinued for 5 Days	2 Months After Stopping TPM
Sodium	141	141	116	116	127	135	142	140
Potassium	4.6	4.2	3.9	3.8	4.2	4.5	4.5	4.3
Bicarbonate	25	30	31	32	26	25	27	27
Chloride	101	101	73	80	90	101	108	104
Creatinine	1	1.1	2.8	3	2.3	2.1	1.7	0.8
BUN	16	16	35	32	24	24	15	10
Glucose	89	64	115	111	110	99	94	91
Calcium	9.6	9.3	9.9	10.1	9.7	9.4	9.8	9.2
Mg	-	-	-	1.8	1.7	1.8	-	1.9
Po4	-	-	-	3.6	3.2	3.9	-	4.5
Uric Acid	-	-	-	12	-	-	-	-

Abbreviation: TPM, Topiramate.

The patient received 100 mL of 3% hypertonic saline in the ED and was then admitted to the medical intensive care unit. Initial management consisted of volume repletion with 0.9% normal saline at 100 mL/hour for 12 hours. Hydrochlorothiazide, lisinopril, and buspirone were discontinued due to electrolyte disturbances and AKI. On hospital day 2, the patient developed rapid correction of hyponatremia to 125 mEq/L and received 2 mcg desmopressin to prevent overcorrection. Renal and bladder ultrasound was unremarkable. On hospital day 3, oral sodium chloride supplementation (2 g thrice a day, or 102 meq Na/day) was initiated and continued until discharge. Scr remained at 2.3 mg/dL, suggesting modest recovery of renal function.

SNa subsequently stabilized between 120-127 mEq/L, and Scr improved but remained elevated at 2.1-2.3 mg/dL through hospital day 4 (Table 1). TPM was discontinued based on clinical suspicion for drug-induced Fanconi-like syndrome.

After TPM discontinuation, clinical improvement was rapid. By hospital day 5, SNa normalized to 135 mEq/L and Scr improved to 2.1 mg/dL. The patient was discharged home. Discharge medications included amlodipine 10 mg daily, lamotrigine 50 mg daily, levetiracetam 750 mg every 12 hours, minoxidil 2.5 mg daily, pantoprazole 40 mg twice daily, propranolol 80 mg daily, sildenafil 100 mg as needed, and zolmitriptan 5 mg twice daily as needed. TPM was permanently discontinued, and the patient's seizure regimen was adjusted accordingly. Outpatient laboratory studies obtained five days after discontinuing TPM demonstrated resolution of hyponatremia (Na 142 mEq/L) and continued improvement in renal function (Scr 1.7 mg/dL; Fig 1). Another 8 weeks later, the Scr normalized (Scr 0.8 mg/dL; Fig 1; Table 1) and the urinalysis showed no glucosuria and the urine pH was 5.5 (Table 2).

**Table 2:** The Change in Urinalysis Before, During, and After Stopping TPM

	113 Months Before Initiation	At Time of Admission on TPM	Day 4 of Hospitalization, TPM Withdrawn After This Test	Day 5 After Stopping TPM	2 Months After Stopping TPM
Urine glucose	Negative	3+	3+	Normal	Normal
Urine pH	6	7	7	6.5	5.5
Urine ketones	Negative	Negative	Negative	Negative	Trace
Urine protein	Negative	Trace	Trace	Negative	Negative
Urine bilirubin	Negative	Negative	Negative	Normal	Normal

Abbreviation: TPM, Topiramate.

## Discussion

This case represents the third reported case of AKI and the second case of AKI and Fanconi.^7^^,^^8^ Expanded TPM administration increases the risk of both common and uncommon medication-related complications. Based on the number of reports, AKI is a rare complication, but a serious complication if unrecognized and not surveilled. Moreover, TPM-associated AKI may be under-recognized because AKI often leads to all medications being discontinued, so the culprit agent may not be identified.

As the second reported case, we sought to identify similarities and differences between the cases to propose possible risk factors associated with TPM related AKI. Both AKI/Fanconi syndrome occurred in subjects administered TPM for more than 1 year; 58 months in the first case and 18 months in our case.^7^ Rapid recognition and discontinuation of TPM resolved the AKI and Fanconi syndrome, implying an excellent prognosis.^7^ Neither case showed any evidence of allergic interstitial nephritis, such as fever, peripheral eosinophilia, or urinary white blood cells, implying a tubulotoxic effect. Each patient had a remote history of smoking tobacco and both had been administered buspirone, concurrently, with TPM. This similarity, use of buspirone and TPM concurrently, seemed quite interesting; however, the literature review did not support any interactions between buspirone and TPM. Buspirone functions as a 5-HT1A partial agonist and a D2 receptor antagonist while TPM acts as a GABA-A receptor activity, and blocks voltage-gated Na channels.^9^, ^10^, ^11^ How these drugs may synergistically function to induce a Fanconi syndrome and AKI is unclear.

On the other hand, differences between the cases exist. Our patient presented with a compensated metabolic alkalosis and severe hypo-osmolal hyponatremia, which is likely related to HCTZ administration. Isotonic saline is the definitive therapy for volume contraction related-metabolic alkalosis, volume depletion mediated hyponatremia, and prerenal azotemia. Initially, there was a robust response to saline administration with improvement in SNa; however, this was quickly tempered by desmopressin injection. At this point the Na stabilized to 122-127 mEq/L for the next 48 hrs with a Scr of 2.3 mg/dL. TPM was discontinued and SNa increased to 130-135 mEq/L and Scr was 2.1-2.2 mg/dL. Though TPM may have contributed to hyponatremia, we suspect the improvement in Na is mostly due to a reduction desmopressin serum level. The half-life of desmopressin in patients with normal renal function is 1.5-3 hours but extends to 6-10 hours in renal failure.^12^ Finally, the original case of AKI/Fanconi, without HCTZ, did not present with hyponatremia.

The exact mechanism underlying the AKI remains unknown. AKI syndromes are due to direct tubular toxicity, hemodynamic mechanisms, intratubular obstruction, or tubulointerstitial nephritis. The AKI in this case is likely due to a combination of volume depletion (hemodynamic) from HCTZ and intrinsic (tubular toxicity) AKI from TPM. Loop and distal tubule diuretics are known to mediate prerenal azotemia associated with metabolic alkalosis. The Scrimproved from the admission value to 3.0 to 2.4 mg/dL, and serum bicarbonate fell from 32 to 28 meq/L after saline infusion, showing saline mediated volume expansion improved Scr and serum bicarbonate. However, at this point, due to the change in SNa, desmopressin was given and saline discontinued. The next day oral NaCl at 102 meq NaCl daily was started until discharge with minimal effect on Scr (2.1-2.3 mg/dL) but normalization of SNa, implying that there may be an intrinsic component to the AKI. The clinical data did not demonstrate evidence of interstitial nephritis, like pyuria, fever, or skin rashes. No evidence exists that TPM can crystallize out of urine and cause AKI. However, five days after stopping TPM, Scr improved to 1.7 mg/dL. Importantly, discontinuation of TPM correlated with normalization of urinary indices, specifically pH and glucosuria. Therefore, the most likely mechanism is direct tubular toxicity since many drugs are associated with Fanconi syndrome and AKI, like tenofovir and cisplatin.^13^

We suspect that these cases may be related to a dose effect. In the first report, the patient had stage 2 chronic kidney disease (60-89 mL/min) and the dosing, based on indication, should have reduced to 50% of those with normal renal function.^14^ In this case, hydrochlorothiazide decreases renal excretion of TPM by ∼25% and increases systemic exposure by nearly 30%.^15^ Therefore, based on these 2 cases, we suspect that TPM-induced AKI/Fanconi syndrome may be due to dose and/or length of exposure since both patients were taking TPM for long periods of time and may have been exposed to a high level of TPM.

The management of this patient followed established principles for drug-induced Fanconi syndrome: immediate discontinuation of the offending agent, supportive care with volume repletion, and electrolyte correction. The rapid clinical improvement in renal function and Fanconi syndrome following TPM discontinuation implies that TPM is likely the causative agent. Our patient's Scr improved to 1.7 mg/dL by one-week post-discharge and returned to normal within 8 weeks after discontinuing TPM (Fig 1). The glucosuria and urine pH also improved over the same time period (Table 2).

Several clinical implications emerge from this case. First, clinicians should maintain a high index of suspicion for proximal tubular dysfunction in patients on TPM who present with unexplained electrolyte abnormalities, particularly if glucosuria is present with normal serum glucose. Second, if TPM renal toxicity is a dose effect, the combination of TPM with thiazide diuretics may increase the risk of AKI and should be monitored closely. Third, while routine monitoring of bicarbonate levels in all TPM-treated patients remains controversial, patients with risk factors such as concurrent diuretic use, renal disease, or other conditions predisposing to acidosis or enhanced TPM half-life may benefit from more frequent periodic assessment. On the other hand, the prognosis for TPM-induced AKI/Fanconi syndrome appears favorable with prompt recognition and drug discontinuation. Our patient's rapid clinical improvement following TPM cessation underscores the importance of early recognition and intervention.

In conclusion, this case illustrates a rare but clinically significant complication of TPM therapy. While TPM-induced metabolic acidosis is common, progression to full Fanconi-like syndrome with severe electrolyte derangements and AKI is uncommon. Clinicians should be aware of this potential complication, particularly in patients receiving concurrent medications that affect TPM half-life or renal function. Prompt recognition and discontinuation of TPM, along with supportive care, typically results in favorable outcomes.
